# Fabrication of Oblique Submicron-Scale Structures Using Synchrotron Hard X-ray Lithography

**DOI:** 10.3390/polym13071045

**Published:** 2021-03-26

**Authors:** Kanghyun Kim, Kyungjin Park, Hyoryung Nam, Geon Hwee Kim, Seong Kyung Hong, Suhyeon Kim, Hyeonsu Woo, Seungbin Yoon, Jong Hyun Kim, Geunbae Lim

**Affiliations:** 1 Department of Mechanical Engineering, Pohang University of Science and Technology (POSTECH), 77 Cheongam-ro, Nam-gu, Pohang, Gyeongbuk 37673, Korea; qwnerkang@postech.ac.kr (K.K.); skhong@postech.ac.kr (S.K.H.); kshyeon@postech.ac.kr (S.K.); whyunsoo@postech.ac.kr (H.W.); ysbin95@postech.ac.kr (S.Y.); 2 School of Interdisciplinary Bioscience and Bioengineering, Pohang University of Science and Technology (POSTECH), 77 Cheongam-ro, Nam-gu, Pohang, Gyeongbuk 37673, Korea; kjpark@postech.ac.kr; 3 Department of Convergence IT Engineering, Pohang University of Science and Technology (POSTECH), 77 Cheongam-ro, Nam-gu, Pohang, Gyeongbuk 37673, Korea; alfzmtea@postech.ac.kr; 4 School of Mechanical Engineering, Chungbuk National University, 1, Chungdae-ro, Seowon-gu, Cheongju-si, Chungcheongbuk-do 28644, Korea; geonhwee.kim@chungbuk.ac.kr; 5 Pohang Accelerator Laboratory (PAL), Pohang University of Science and Technology (POSTECH), 77 Cheongam-ro, Nam-gu, Pohang, Gyeongbuk 37673, Korea

**Keywords:** X-ray lithography, oblique gold absorber, oblique sub-micron structure, contrast, double exposure

## Abstract

Oblique submicron-scale structures are used in various aspects of research, such as the directional characteristics of dry adhesives and wettability. Although deposition, etching, and lithography techniques are applied to fabricate oblique submicron-scale structures, these approaches have the problem of the controllability or throughput of the structures. Here, we propose a simple X-ray-lithography method, which can control the oblique angle of submicron-scale structures with areas on the centimeter scale. An X-ray mask was fabricated by gold film deposition on slanted structures. Using this mask, oblique ZEP520A photoresist structures with slopes of 20° and 10° and widths of 510 nm and 345 nm were fabricated by oblique X-ray exposure, and the possibility of polydimethylsiloxane (PDMS) molding was also confirmed. In addition, through double exposure with submicron- and micron-scale X-ray masks, dotted-line patterns were produced as an example of multiscale patterning.

## 1. Introduction

Nanostructured surfaces of various shapes have been studied for use in various types of research, such as for cell-behavior platforms [1,2], anti-reflective surfaces [3,4], and artificial-wetting surfaces [5,6,7]. It is remarkable that the properties of nanostructures can be controlled by designing their shape. For example, tilted shapes can be used to create asymmetric surface properties. [8,9]. The van der Waals force of gecko setae is a key area of dry-adhesive research. Due to the oblique nanostructure of the setae, adhesion can be released when force is applied in the opposite direction, unlike typical glues [10]. In addition, oblique nanostructures can be used to modify the directional wettability of surfaces. Such surfaces allow transport control in various fluid systems and have been demonstrated in oblique structures at the scale of a few hundred nanometers [11,12,13].

Several of the deposition and etching techniques of lithography have been employed to fabricate oblique submicron patterns. Glancing angle deposition (GLAD) is a representative method that is used to fabricate oblique structures with various materials. After initial nucleation on the substrate, deposition proceeds, allowing nanocolumns to grow from the nuclei; the angle of the nanocolumns is determined by the deposition angle. Electron beam evaporation, which is widely used for deposition, is useful for producing SiO_2_ structures [14]. In addition, physical vapor deposition (PVD) and plasma-enhanced chemical vapor deposition (PECVD) have been used to fabricate Ag/Alq3 and ZnO/Alq3 hybrid structures, respectively [15]. However, it is difficult to control the structure of individual lines accurately [16]. Plasma etching, which is typically used to create vertical patterns, has been studied as a way to build tilted structures by guiding the path direction of the etching species. For such guidance, the potential distribution can be changed by placement of a glass block. However, this results in the angle having a nonlinear relation with the location [17]. In contrast, in another method, the tilted potential distribution is straightened using a Faraday cage for the slanted sample. This has the disadvantage that it requires the addition of an etching mask deposition process, and the complexity of the system increases due to the additional setup [18]. Electron beam lithography has also been introduced and was used to produce a 45° tilted hole-and-pillar array with a diameter >100 nm [19]. This method has high resolution, but it is time consuming for use over a large area because it is a direct-writing technique. In addition, oblique nanostructures have been fabricated using a combination of lithography, etching, and transfer [20,21].

Here, we propose a simple and angle-controllable method based on X-ray lithography to fabricate oblique submicron-scale structures with areas at the centimeter scale. Because the wavelength of X-rays is very short, diffraction is sufficiently small to form submicron-scale areal images in the photoresist; however, an oblique submicron-scale X-ray mask is not easy to fabricate. Therefore, an oblique submicron X-ray mask, which was fabricated by thermal deposition of gold on the slanted wall, was designed by improving the vertical submicron scale X-ray mask [22]. With this X-ray mask, oblique X-ray exposure was conducted.

## 2. X-ray Mask Fabrication

### 2.1. Design Principle of the X-ray Mask with Oblique Sub-Micron Structures

**Figure 1:**
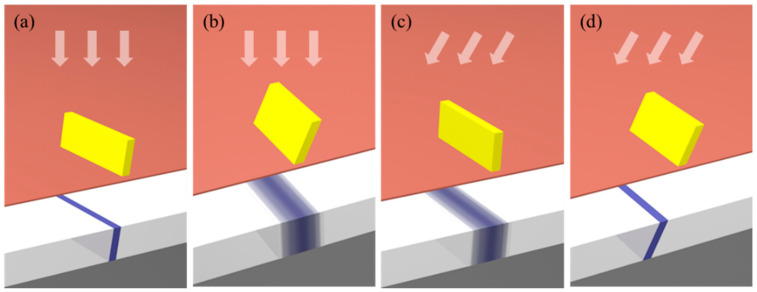
Schematic diagram of transient dose region suppression. White arrow indicates the incident angle of X-rays, yellow strip is the absorber and the blue line is the exposed region. When X-rays were incident to normal direction, (**a**) the vertical absorber had a narrower transient dose region than (**b**) the oblique absorber. In the oblique exposure, (**c**) the vertical absorber had a broad transient dose region, and (**d**) the oblique absorber had a narrow region.

To form images in a resist, a mask is used in X-ray lithography, as in photolithography. The X-ray mask is composed of a region that is transparent to X-rays and an absorber made of heavy metal. Sufficient image contrast between the two parts must be guaranteed because this affects the patterning quality. In our system, polyimide (PI) was chosen for the transparent part due to its low absorption of X-rays and chemical stability. The absorber layer was typically several-micrometer-thick gold, as a high-Z (atomic number) material. In addition, in oblique X-ray lithography, tilted gold absorber structures are desirable for precise alignment that minimizes the transient dose region [23,24]. In the typical exposure scheme, a vertical absorber allows the dose distribution to be well defined, as shown in Figure 1a. However, as shown in Figure 1b, misalignment widens the distribution. On the other hand, in oblique X-ray exposure, the dose distribution caused by the vertical absorber (Figure 1c) is broader than that caused by the oblique absorber (Figure 1d). Therefore, for the fabrication of inclined nanostructures, a novel X-ray mask was designed using patterning of a tilted micron-scale backbone with typical UV lithography together with a subsequent metal-deposition process on the inclined surface of the backbone. In this way, the thickness of the absorber can be equivalent to the thickness of the backbone pattern.

### 2.2. Fabrication of the Sub-Micron Scale X-ray Mask

The negative-type epoxy photoresist SU-8 that is commonly used in UV lithography was selected as the backbone material because it shows high transmittance and durability in response to X-rays. The backbone was designed with 15-mm-long, 5-μm and 10-μm periodic 1:1 line patterns with a width of 15 mm. SU-8 3005 (Kayaku Advanced Materials Inc, Westborough, MA, USA) was spin-coated at 5000 rpm onto the PI film, which was ~4 µm thick. The soft bake-out process was performed on a hotplate at 95 °C over a period of 2 min. The mask and substrate were brought together using a vacuum and tilted 10° or 20° according to the target angle; the SU-8 photoresist was exposed to UV with a wavelength of ~365 nm at a dose of 105 mJ/cm^2^. The post-bake-out process was performed for 1 min sequentially at 65 °C and 95 °C, and the development process proceeded for 1 min in a dedicated SU-8 developer (Kayaku Advanced Materials Inc, Westborough, MA, USA). Finally, inclined micron-scale line patterns with a parallelogram shape in cross-section were formed.

**Figure 2:**
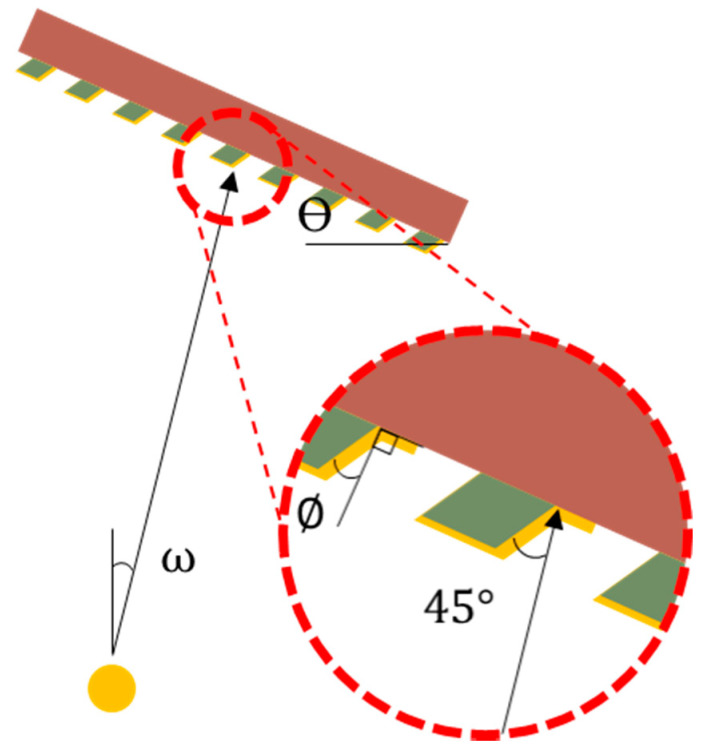
Scheme of the gold absorber deposition on the side wall.

**Figure 3:**
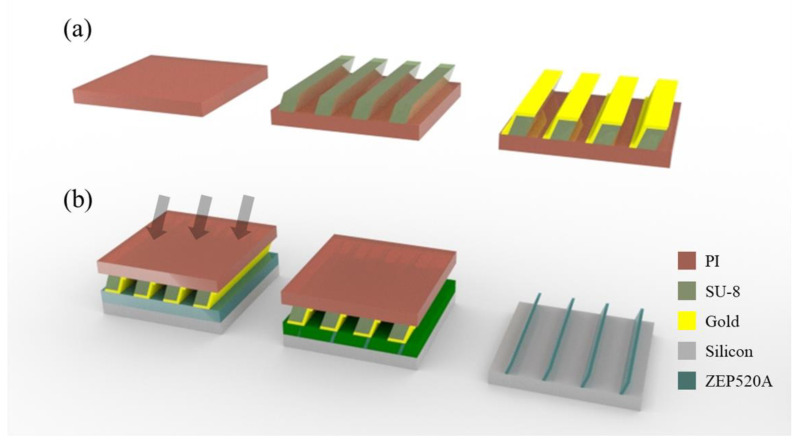
Fabrication process of the oblique sub-micron (**a**) X-ray mask and (**b**) line structure using X-ray lithography.

The absorber was deposited on the slanted backbone by thermal deposition. The geometry of the thermal deposition components determines the deposited thickness. Gold pellet (iTASCO, Seoul, Korea) 6 g was heated in a tungsten boat (iTASCO, Seoul, Korea). The set sample loading angle, θ, of the wafer was as shown below:

(1) θ=ω−∅+45

where ω is the traveling angle of gold related to normal direction to ground, and ∅ is the target angle of the backbone. In this experiment, the angle between the backbone wall and the deposition direction was set to 45° for the entire process of deposition on the sidewall. The angle between the gold source and the sample was 11°, and the loading angles of the 70° and 80° samples were 36° and 46°, respectively. The deposition scheme is depicted in Figure 2. The mask fabrication process and the usage of the mask are summarized in Figure 3.

## 3. X-ray Exposure Experiment for the Oblique Sub-Micron Structure

### 3.1. Intensity Contrast Calculation

**Figure 4:**
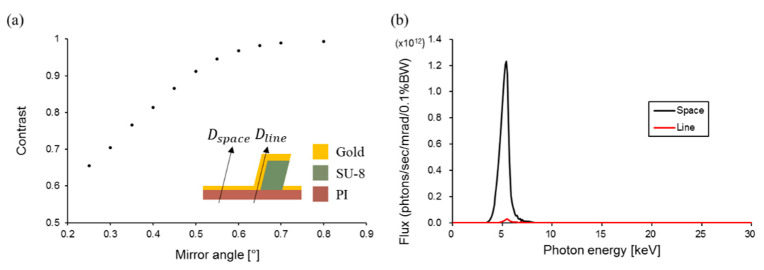
(**a**) Contrast according to the mirror angle (Arrows are dose on space ($D_{space}$) and dose on line ($D_{line}$)) and (**b**) spectra difference from the mirror set 0.6° between the line and the space.

In X-ray lithography, higher contrast intensity is associated with more precise patterns. The contrast is calculated using the equation below:

(2) C= (Imax−Imin)(Imax+Imin)=(Dspace−Dline)(Dspace+Dline)

where $I_{max}$ and $I_{min}$ are the maximum and minimum intensity, respectively, which can be replaced by the dose on space, $D_{space}$ and dose on line, $D_{line}$, respectively; dose on space is the transmittance of the gold deposited at a thickness of 0.5 µm on the bottom of the mask, and dose on line is the transmittance of 4-μm-thick gold. The contrast is related to the mask design and the spectral range of the source energy, which can be modulated using optics. In this study, the bending magnet of the 9D X-ray nano/micromachining (XNMM) beamline in the Pohang Accelerator Laboratory was used as an X-ray source. The energy spectral range of the beamline can be modulated by adjusting the angle of a pair of total-reflection X-ray mirrors. Contrast is improved at higher mirror angles by the cutoff in intensity in the high-energy region that is not readily absorbed. The calculated contrast depending on the mirror angle is shown in Figure 4a. When the mirror was set to 0.6°, the contrast was 0.968 with 20 min of exposure time, and detailed spectra are shown in Figure 4b.

### 3.2. Fabrication of Oblique Sub-Micron Structures

Positive ZEP 520A was selected as an X-ray resist due to its high sensitivity. ZEP 520A was spin-coated on the silicon wafer at 500 rpm and baked for 3 min at 180 °C to achieve a thickness of ~0.7 µm. In addition, one further coat of ZEP 520A was applied at a thickness of 1.5 µm for high-aspect-ratio patterns. The sample was loaded onto an aluminum jig, and the fabricated X-ray submicron mask was attached to the sample. The jig was fixed on the stage under vacuum and aligned with the X-ray angle of incidence. The single-coated PR was inclined at 10° and exposed to X-rays at a dose of 1.3 kJ/cm^3^. The double-coated PR was inclined at 20° and exposed to X-rays at a dose of 1.5 kJ/cm^3^ with helium. Following exposure, the development process was performed in ZED N50 developer for 30 s, followed by rinsing in the mixture of methyl isobutyl ketone and isopropyl alcohol (89:11 *v*/*v*) for 10 s.

**Figure 5:**
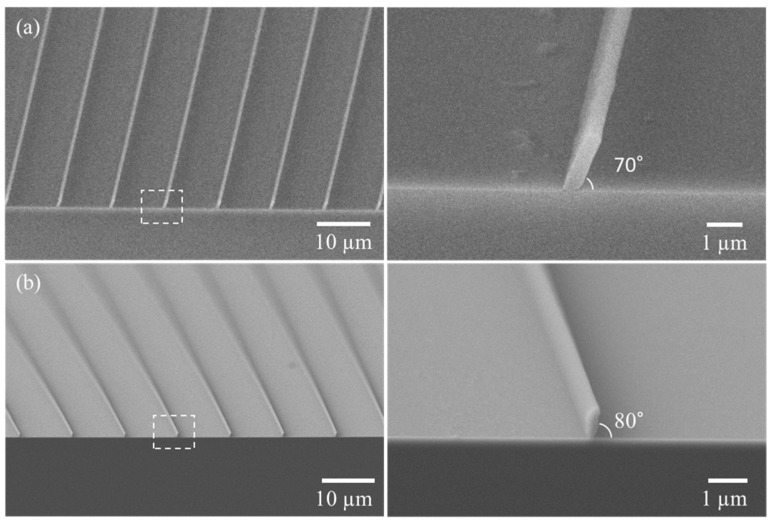
Oblique sub-micron structures and the magnified single structure of 10 µm period at (**a**) 20°, (**b**) 10°.

The angle of the ZEP 520A photoresist was measured on cross-sectional images obtained by scanning electron microscopy (SEM, JSM-7401F, JEOL LTD, Akishima, Tokyo, Japan). The angles of the pattern were the same as the objective angles of the mask. The line width of the 20° sample was ~510 nm, and it had an aspect ratio of ~2.94. The 80° sample had a line width of 345 nm and an aspect ratio of 2.03, as shown in Figure 5a,b. The pattern length was equivalent to the period of the mask under both conditions.

### 3.3. Fabrication of Oblique Groove Structures

**Figure 6:**
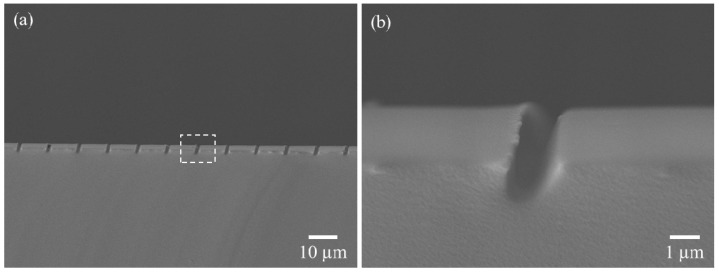
(**a**) Periodic molded groove PDMS patterns at 20°, and (**b**) the shape of the groove.

In addition, to fabricate groove structures, polydimethylsiloxane (PDMS) soft lithography was conducted instead of direct patterning on a negative resist. The PDMS base and the curing agent (Dow Corning, Midland County, MI, USA) were mixed at 10:1 (*w*/*w*) and cured overnight in an oven at 60 °C (OV-11; Jeio Tech, Daejeon, Korea). It was confirmed that the slanted PDMS groove had the same depth and period as the mold based on SEM, as shown in Figure 6. However, the width and angle differed slightly from the patterned mold, which was inferred to be due to the high aspect ratio of the oblique structure and the resolution of the PDMS.

### 3.4. Fabrication of the Multi Scale Structure Using Double Exposure

Using a combination of a nano X-ray mask and a general micron-scale X-ray mask, unique structures, including oblique dotted-dashed line structures, were manufactured. To realize such fusion structures, a typical micron-scale X-ray mask for the manufacture of micrometer-scale devices with high aspect ratios was produced using UV lithography and gold electroplating. A 100-nm-thick gold layer was deposited on a 200-μm-thick PI film as the substrate. The X-ray-transparent part was formed using negative photoresist SU-8, and the other absorber part was plated with 4-μm-thick gold [22]. The design was 50-μm-wide lines spaced at 200 µm.

**Figure 7:**
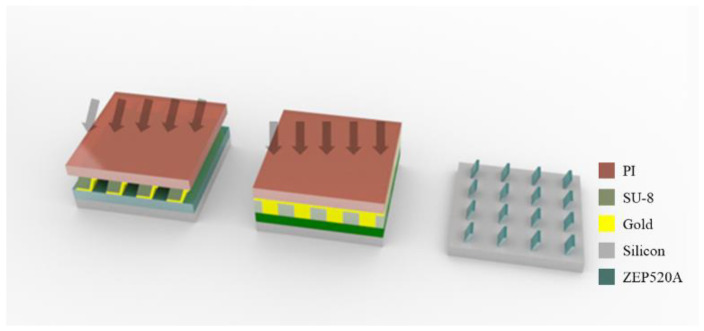
Fabrication of multi-scale structures with a dotted–dashed line shape. Oblique exposure for sub-micron structure was followed by vertical exposure for micron structure. Final structure was periodic sub-micron oblique structure.

To implement the fusion patterns, dotted lines were fabricated through double exposure. After the first oblique exposure using the abovementioned nano-scale X-ray mask, the second exposure using the micron-scale X-ray mask proceeded in the normal direction using the same exposure dose. Using this process, a 20° fusion pattern was fabricated, as shown in Figure 7.

**Figure 8:**
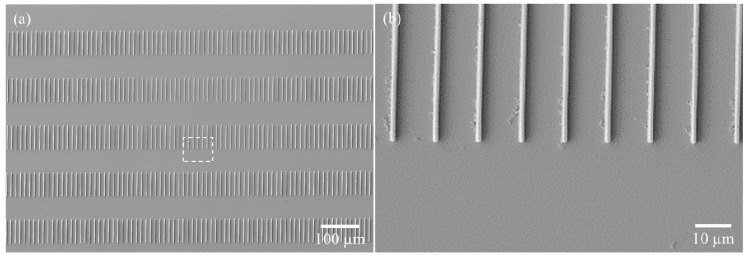
(**a**) 1-to-1 line length and space exposed by the micron scale mask and (**b**) the shape of each line in the oblique fusion structures.

**Figure 9:**
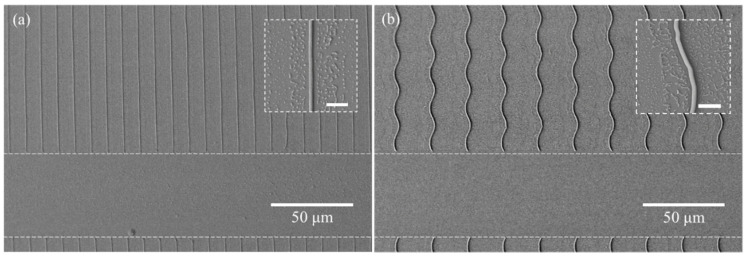
Oblique long dotted (**a**) linear and (**b**) wavy line structures. (Scale bar in the small box = 3 µm).

The dotted-dashed line length and spacing can be controlled by changing the fill factor of the micron-scale mask. In addition, oblique fusion structures of various shapes with different lengths or periods can be realized using this technique. As examples, short periodic line patterns were fabricated using a micron-scale mask for a 1:1 line and spacing pattern (Figure 8). The oblique nano-scale mask can also be designed to produce wavy lines. Using the same process, wavy lines in addition to straight lines were also created successfully (Figure 9).

## 4. Conclusions and Discussion

We developed simple and angle controllable X-ray lithography method and produced oblique submicron-scale structures of ZEP 520A photoresist with areas at the centimeter scale. The engraved PDMS pattern was also produced using soft lithography. A submicron-scale X-ray mask was designed to maximize the contrast considering the beamline characteristics, and a robust thin absorber was produced by the oblique deposition method. Therefore, high-aspect-ratio 20° and 10° slanted patterns were produced that were 1.5 µm and 0.7 µm thick, respectively. Thus, the angle and thickness can be adjusted. In addition, using the double-exposure method with a micro-scale X-ray mask, the possibility of fusion patterning was confirmed for patterns including dotted lines and spacing.

The developed novel method has the advantage of achieving a high aspect ratio, precise oblique angles, and a relatively large patterning area without requiring a complex system. Due to the flexibility of the X-ray mask design, pattern characteristics such as shape, length, and period can be easily controlled. This technology can be combined with other manufacturing techniques to produce hierarchical structures. Therefore, it is expected to contribute to the development of devices with asymmetric nano-scale phenomena, such as dry adhesives and hydrophobic surfaces.

## Data Availability

The study did not report any data.
